# Inhaled nitric oxide: an sGC-dependent IOP lowering agent

**DOI:** 10.1186/2050-6511-16-S1-A38

**Published:** 2015-09-02

**Authors:** Wolfgang S Lieb, Stefan Munster, Ana C Dordea, Sara Vandenwijngaert, Robert E Tainsh, Peter Brouckaert, Warren M Zapol1, Emmanuel S Buys

**Affiliations:** 1 Department of Anesthesia, Critical Care, and Pain Medicine, Massachusetts General Hospital and Harvard Medical School, Boston, 02114, MA, USA; 2 Department for Biomedical Molecular Biology, Ghent University, Ghent, 9000, Belgium

## Background

The nitric oxide (NO)-soluble guanylate cyclase (sGC)-cyclic guanosine 3’5’-monophosphate (cGMP) pathway regulates intraocular pressure (IOP). Preclinical and clincial studies have demonstrated the ability of NO-donor compounds to lower IOP (e.g. VESNEO^®^). The use of inhaled NO gas (iNO), a specific pulmonary but not systemic vasodilator, is an approved therapy for pulmonary hypertension and is under development as a treatment for other cardiovascular diseases (e.g. for myocardial ischemia, the NOMI trial). We hypothesized that breathing NO lowers IOP in an sGC-dependent manner.

## Methods

*Anesthetized IOP model:* 10- to 20-week-old male wild-type (WT) mice and mice deficient in the α1-subunit of sGC (sGCα1^-/-^ mice; n=9, each) were anesthetized with isoflurane using a standard protocol resulting in a stable IOP baseline. Ten minutes after baseline measurement, IOP was measured again in mice breathing 1.8 % isoflurane and either control gas (N_2_ balanced in O_2_) or 40 ppm NO balanced in O_2_. *Awake IOP model:* WT mice were acclimated to awake IOP measurements (every other day for 2 weeks). 40 min after baseline measurements, IOP was measured in mice breathing either control gas or 40 ppm iNO (n=8, each) in an incubation chamber.

## Results

**Figure 1:**
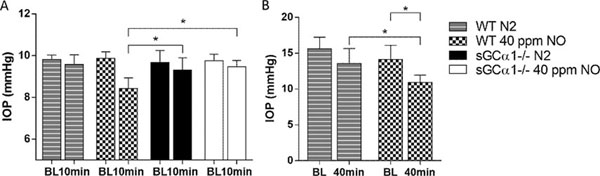
iNO decreases IOP in WT but not sGCα1^-/-^ mice:

Breathing control gas did not affect IOP in WT or sGCα1^-/-^ mice (*Figure*1). Breathing iNO decreased IOP in both anesthetized WT mice (9.86±0.31 vs. 8.42±0.51 mmHg at baseline and after iNO, respectively, Figure 1a) and awake WT mice (14.13±1.95 vs. 10.93±1.01 mmHg, at baseline and after 40 min iNO, respectively, Figure 1b). In contrast, iNO did not lower IOP in sGCα1^-/-^ mice (9.75±0.31 vs. 9.46±0.30 mmHg at baseline and after iNO, respectively, Figure 1a).

## Conclusion

Inhalation of 40 ppm iNO decreased IOP in anesthetized and awake WT mice but not in sGCα1*-/-* mice. These findings confirm that NO is an IOP-lowering agent, and identify NO-gas as a possible therapeutic approach to acutely lower IOP. In addition, our results identify sGC as the downstream target of NO's ability to lower IOP. sGC stimulators, under development for treatment of cardiovascular diseases, such as the recently approved ADEMPAS^®^, may be considered as a novel treatment option for elevated IOP.

